# Introducing chaotic codes for the modulation of code modulated visual evoked potentials (c-VEP) in normal adults for visual fatigue reduction

**DOI:** 10.1371/journal.pone.0213197

**Published:** 2019-03-06

**Authors:** Zahra Shirzhiyan, Ahmadreza Keihani, Morteza Farahi, Elham Shamsi, Mina GolMohammadi, Amin Mahnam, Mohsen Reza Haidari, Amir Homayoun Jafari

**Affiliations:** 1 Medical Physics & Biomedical Engineering Department, School of Medicine, Tehran University of Medical Sciences, Tehran, Iran; 2 Research Center for Biomedical Technologies and Robotics (RCBTR), Tehran University of Medical Sciences, Tehran, Iran; 3 Section of Neuroscience, Department of Neurology, Faculty of Medicine, Baqiyatallah University of Medical Sciences, Tehran, Iran; 4 Department of Biomedical Engineering, Faculty of Engineering, University of Isfahan, Isfahan, Iran; School of Psychology, CHINA

## Abstract

Code modulated Visual Evoked Potentials (c-VEP) based BCI studies usually employ m-sequences as a modulating codes for their broadband spectrum and correlation property. However, subjective fatigue of the presented codes has been a problem. In this study, we introduce chaotic codes containing broadband spectrum and similar correlation property. We examined whether the introduced chaotic codes could be decoded from EEG signals and also compared the subjective fatigue level with m-sequence codes in normal subjects. We generated chaotic code from one-dimensional logistic map and used it with conventional 31-bit m-sequence code. In a c-VEP based study in normal subjects (n = 44, 21 females) we presented these codes visually and recorded EEG signals from the corresponding codes for their four lagged versions. Canonical correlation analysis (CCA) and spatiotemporal beamforming (STB) methods were used for target identification and comparison of responses. Additionally, we compared the subjective self-declared fatigue using VAS caused by presented m-sequence and chaotic codes. The introduced chaotic code was decoded from EEG responses with CCA and STB methods. The maximum total accuracy values of 93.6 ± 11.9% and 94 ± 14.4% were achieved with STB method for chaotic and m-sequence codes for all subjects respectively. The achieved accuracies in all subjects were not significantly different in m-sequence and chaotic codes. There was significant reduction in subjective fatigue caused by chaotic codes compared to the m-sequence codes. Both m-sequence and chaotic codes were similar in their accuracies as evaluated by CCA and STB methods. The chaotic codes significantly reduced subjective fatigue compared to the m-sequence codes.

## Introduction

Visual evoked potentials (VEPs) are EEG responses to the visual stimuli. Brain-computer interfaces (BCI) based on these potentials are becoming popular, for their less training time and high information transfer rate (ITR) [1]. VEP-based BCI systems can be classified into three different categories: time modulated, frequency modulated and code modulated stimuli [2]. In systems with the time modulated stimuli, the sequence of target stimuli is coded in non-overlapping time windows such as P300 based BCI system. This, however, usually leads to low ITR [2]. In systems with frequency modulated stimuli, different targets are defined by their distinct frequencies that can be recognized by detecting the same target frequencies and their harmonics [2] and phase information of the evoked responses [3, 4]. In code modulated BCI systems, the pattern of flashing is determined by using a pseudo-random manner sequence such as an m-sequence [5]. In this modality the work mechanism is based on using the different shifts of modulating codes. These codes have Dirac like auto-correlation function that allows using shifted versions of modulating codes as different targets for evoking different VEPs. A simple and short calibration allows to have a specific EEG response to the m-sequence, and with that, all the targets that are lagged versions of the same m-sequence can be distinguished [2, 6].

The signals transmitted via broadband codes lead to robustness to noise and lower cross interferences of other stimuli because the auto-correlation of broadband code exhibits Dirac function [7].

Code modulated Visual Evoked potentials (c-VEPs) utilize characteristics of broadband codes as stimuli. Broadband codes have the capability of evoking the VEPs that have the appropriate auto and cross-correlation properties [8, 9]. c-VEP based BCIs could play an important role in better system performance and target identification. These also give low cross interference when high number of commands are presented simultaneously leading to significantly high ITR [10].

c-VEP evoked by different lags of non-periodic binary codes could be demodulated in brain responses such as EEG with template matching [6]. High ITR in c-VEP based BCI applications has been achieved by using canonical correlation analysis (CCA) and template matching [11]. Utilizing m-sequence code, c-VEP based BCI has been used to build a BCI system for amyotrophic lateral sclerosis (ALS) patients with significantly higher communication rate only with eye gaze [12]. It has been successfully tested in online applications such as spelling [13] with error-related potential and unsupervised learning for online adaptation and continues to be employed in the control of mobile robots [14, 15].

Novel paradigms for c-VEP based BCIs include the introduction of the generative framework for predicting the responses to gold codes [16], spatial separation and boundary positioning for decoupling of responses to different targets [17]. In addition, target identification in c-VEP based BCIs has been improved by using Support Vector Machine (SVM) method and accuracy has been increased with linear kernel [18]. However, more recently spatiotemporal beamforming (STB) method was used for target identification in c-VEP responses and was found to be significantly better than SVM [9]. Additional measures in this regard include optimization of the stimuli presentation parameters such as color and size of LED, code length, stimuli proximity, and the lag between stimuli [19] and use of dry electrodes [20]. Recent studies in c-VEP based BCIs have increased selectable targets by using different pseudo-random codes such as m-sequence code that have low cross-correlation value with each other [21, 22].

Chaos has been widely observed in various biological systems [23–25]. Chaotic behavior has also been observed in several neuronal structures such as cells, synapses [26, 27], and neural networks [28–32]. Chaotic dynamics has been attributed to large scale brain activities and physiological processes [33] such as information processing [34], synaptic plasticity [35], memory [36], perceptual processing and recognition of unknown odor [37] and also brain state transitions [38].

While randomness has been observed in various aspects of neural system such as rapid random fluctuation in membrane [39], spontaneous activity of neurons [40] and neural spiking [41], however, non-randomness, nonlinearity and chaotic dynamics exists at all levels of brain function from the simplest up to the complex systems [42–47]. In summary, chaos provides the ability of reacting adaptively to outside world leading to new patterns and fresh ideas and contributes to the complex behavior in the brain functions [48–50].

Nonlinear and chaotic dynamics of neural activities also manifest themselves in EEG signals [51–56]. Nonlinear and chaotic analyses methods have been utilized in EEG signal processing [57], feature extraction and analysis in BCI applications [58–60]. Deviation from the normal chaotic behavior of EEG signals is observed in neurological disorders [61, 62] such as epileptic seizures [62–64], depression [65, 66], Alzheimer’s disease [53, 67] and Autism [68]. However, so far there is no study in BCI applications that has employed chaotic code in visual stimulation.

Reduction of visual fatigue has been a challenging issue in VEP based BCI applications [69–71]. Continuous exposure to changes in luminance is highly uncomfortable for the users gazing it [72]. Therefore, designing the stimuli that cause less visual fatigue and discomfort could be valuable in designing a suitable and ergonomic BCI setup. An efficient encoding of visual pattern with low discomfort level occurs when images or flicker have statistical characteristics of natural sense and are more close to 1/f spectral property in temporal or spatial frequency [73–75]. Interestingly, the spectrum of chaotic behavior are reported to be close to the 1/f spectral property [76, 77] as mostly seen in natural scenes and phenomena. As m-sequence codes have the inherent property of random process with flat spectrum [78, 79], using chaotic codes generated with nonlinear dynamical system for reducing visual discomfort are superior to m-sequence.

Employing chaotic behavior to generate codes in spread spectrum communication is taken into consideration from the chaotic maps that provide an infinite number of uncorrelated signals with great correlation properties [80] and suitable for Code Division Multiple Access (CDMA) modulation applications [81, 82]. Use of complimenting binary chaotic sequence also helps in generating broad band chaotic code [83]. As a result the chaotic codes have high correlation property, and using them can lead to high accuracies as m-sequences.

Despite the suitability of chaotic codes for use in c-VEP based BCI applications, so far they have not been used as visual stimuli for c-VEP generation. Therefore, in this study we used chaotic codes and widely used m-sequence codes to evoke c-VEPs in EEG signals and compared their accuracy using CCA and STB methods. In addition, we used VAS to compare subjective fatigue rates between these two codes in normal subjects.

## Material and method

### Study participants

Forty-Four volunteers (21 females), aged 20–33 years old (26.09 ± 3.67) with normal or corrected vision to normal (6/6) participated in this study. The subjects informed via announcement based on the notice boards of the faculties of medicine and biomedical engineering and word of mouth. Subjects with a history of visual or neurological disorders, head trauma or use any drugs that would affect nervous system function were excluded. Before the experiment began, participants signed written informed consent form and the total procedure of signal recording and experiment was described to them. The experimental protocol was approved by the office of research review board and the research ethics committee of the Tehran University of Medical Sciences.

### Experimental design

#### Stimuli

In this study, we used 31-bit m-sequence code that is commonly used in c-VEP based BCIs for its favorable correlation property [2] and is used in other medical fields such as studying visual receptive fields properties [84] and fMRI [85]. We generated 31-bit chaotic code using the logistic map with good auto-correlation property [83] that makes it suitable for using in CDMA based BCIs by the algorithm described as follow, bit ‘0’ presented with dark and bit ‘1’ presented with light stimulation.

#### Generation of chaotic code using logistic map

**Fig 1:**
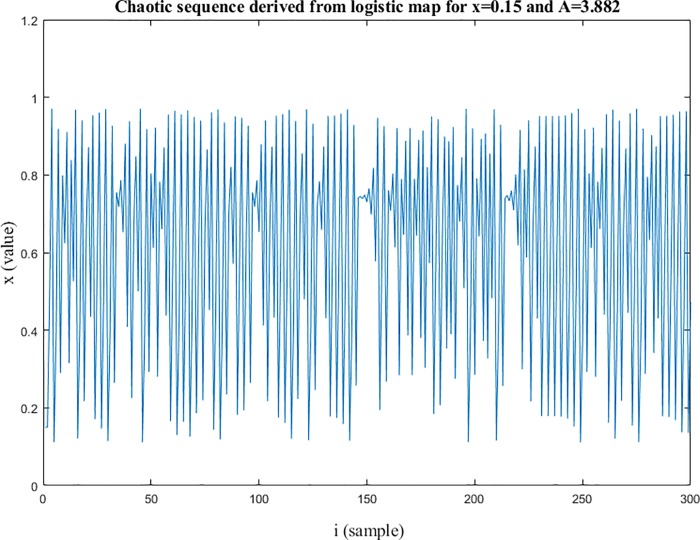
The chaotic sequence. The sequence derived from logistic map for *x* (0) *=* 0.15 and *A =* 3.882.

Chaotic signals have the potential of designing codes that have auto-correlation close to Dirac like function so that correlation method performs well in the identification and makes it appropriate for code modulating applications [86]. The logistic map is a one-dimensional map that can most of natural phenomena and population growth of biological species [87], as defined in (1). Where *x* is in the interval of [0 1] and indicates the ratio of existing population to the maximum possible population, the *x*(0) as the initial value of *x* and *A* is the rate for reproduction and starvation that is at the interval of [0 4]. This simple map could generate chaotic dynamic in some values of parameter *A* generally between 3.5 to 4 [88]. An example of chaotic sequence generated from logistic map is shown in Fig 1 for *A* parameter equal to 3.882 and initial value *x*(0) equal to 0.15.

x(i+1)=Ax(i)(1−x(i)) (1)

**Fig 2:**
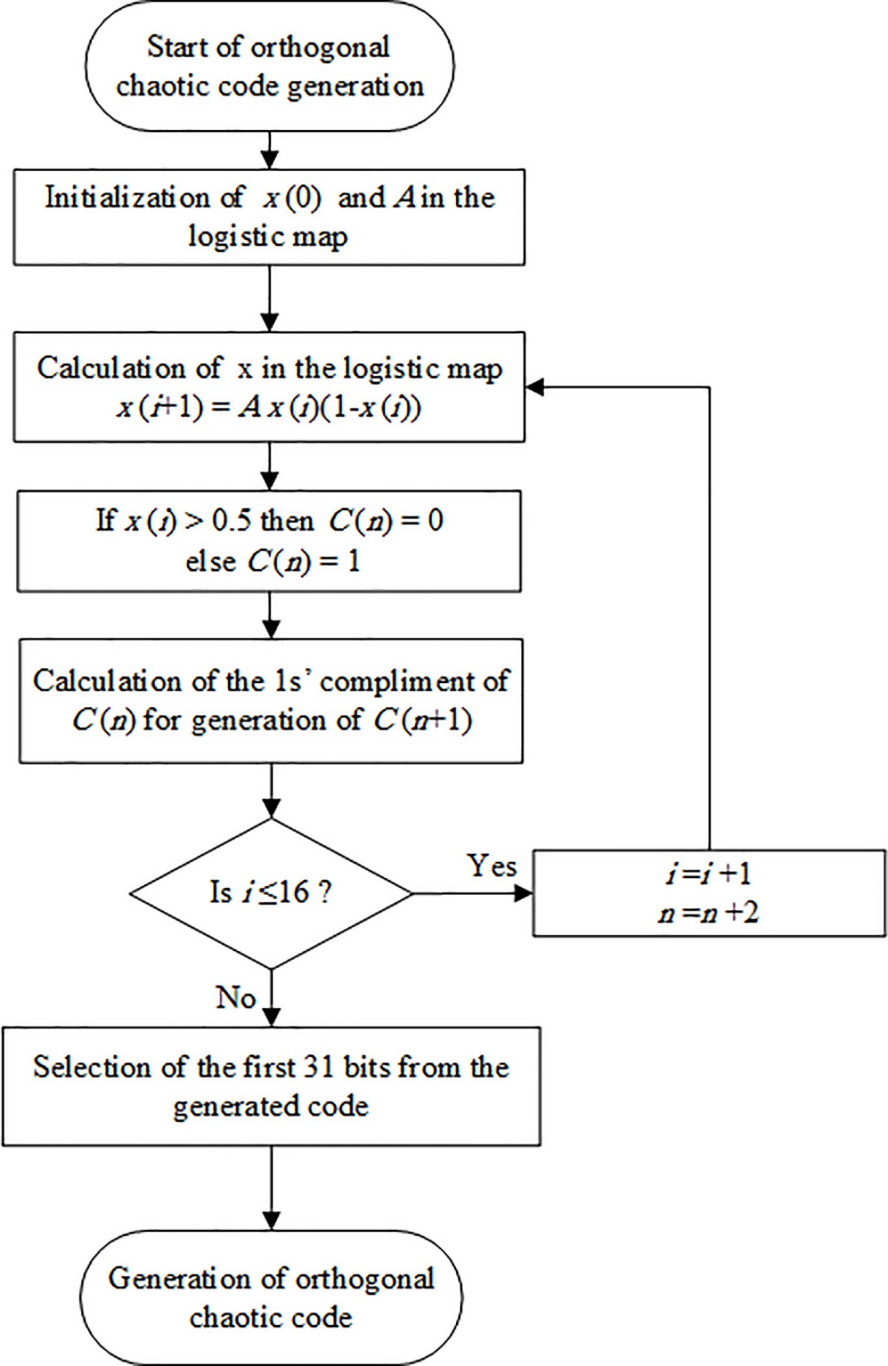
Flowchart for the generation of orthogonal chaotic code.

The algorithm of chaotic code generation is as follows and shown in Fig 2.

1. Selection of the initial value *x*(0) and *A* parameter in Eq 1, (we chose *x*(0) *=* 0.015 and *A* = 3.882)
2. Calculation of the *x*(*i+*1) from the (1).
3. Generation of binary code from *x*(*i*+1):
  If *x*(*i+*1)>0.5 then *C*(*n*) = 0 else *C*(*n*) *=* 1.
4. Taking the 1’s complement of *C*(*n*) to generate *C*(*n*+1) *= C’*(*n*).
5. Checking the condition (*i*≤16). If it is satisfied then increase *i* by 1 and *n* by 2 and then proceed to the step 2, if it is not satisfied, proceed to the next step.
6. Selection of the first 31 bits from the generated code.

**Fig 3:**
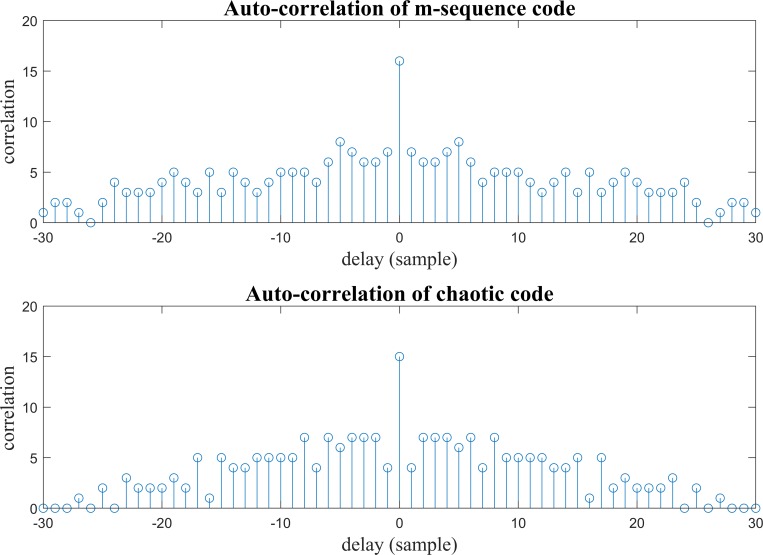
**The auto-correlation of m-sequence code (top) and chaotic code (bottom).** The generated chaotic code follows the correlation property which is necessary in code modulation.

The auto-correlation of m-sequence and chaotic codes are shown as function of delay in Fig 3, here the delays of codes are according to samples (bits) of codes. It can be seen that the auto-correlation of m-sequence code and generated chaotic code are almost Dirac like function, so that the generated chaotic code by the proposed algorithm could be appropriate to be used in the code modulation.

**Fig 4:**
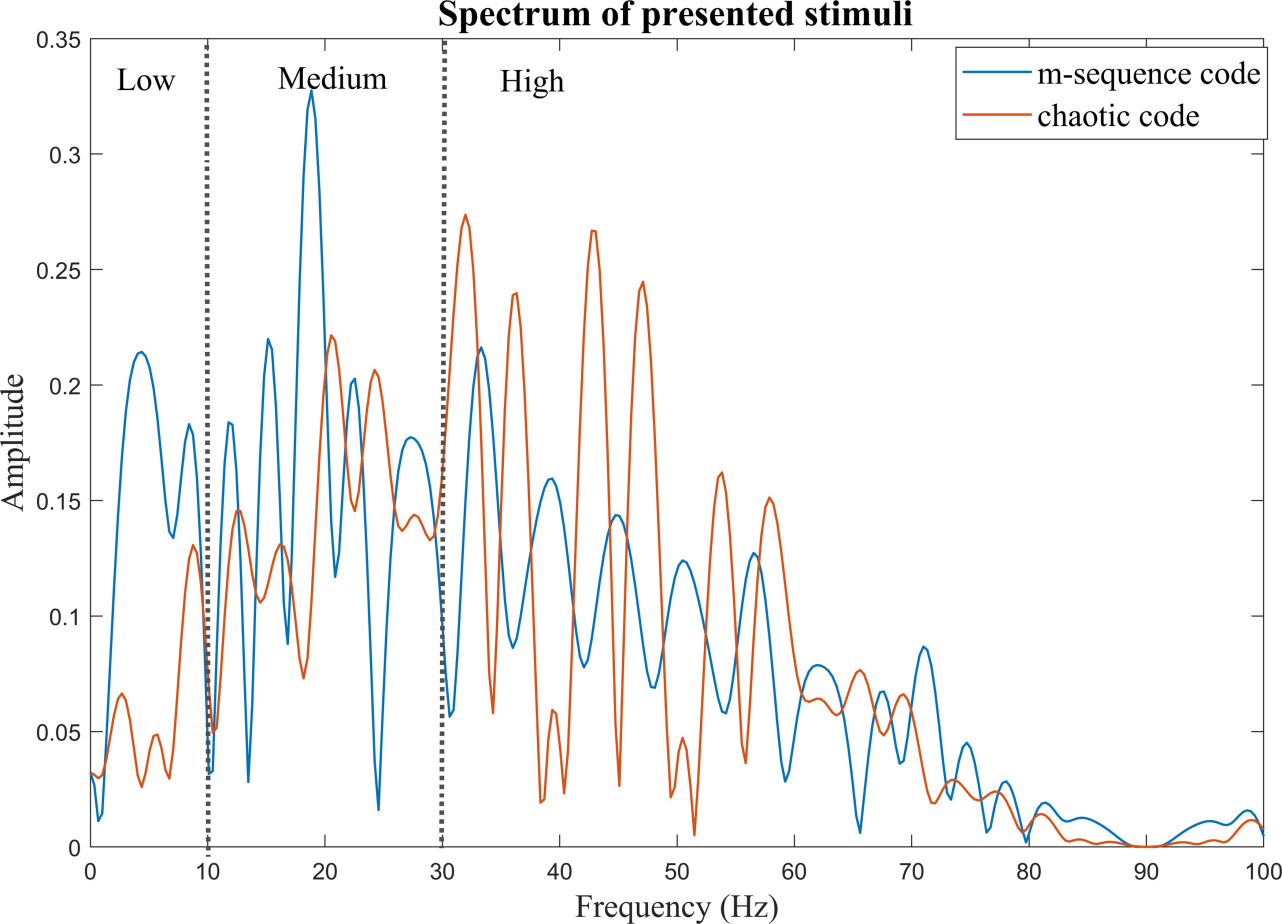
One-sided amplitude spectrum of the presented stimuli (blue: Spectrum of the m-sequence codes stimuli, red: Spectrum of the chaotic code stimuli). Dashed lines separate Low, Medium and High frequencies. Low: frequencies from 0 to 10 HZ, Medium: frequency range between 10 to 30 Hz and High: frequencies above 30 Hz. It is obvious that compared to the m-sequence codes, the chaotic codes frequency components are less in Low and Medium frequencies and are more in High frequency range.

The one-sided amplitude spectrum of presented stimuli of m-sequence and chaotic codes stimuli are shown in Fig 4. Both of the stimuli are broad band. The dashed lines separate the low, medium and high frequency regions. Significant peaks of the m-sequence code are seen in low and medium frequencies. For the chaotic codes, the spectrum of stimuli shows dominant peaks in high frequencies components.

#### Stimuli presentation paradigm

**Fig 5:**
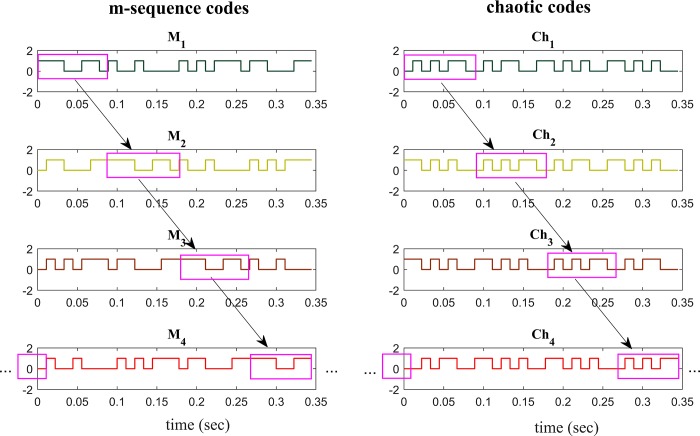
Time domain representation of m-sequence and chaotic codes. Left and right columns show the m-sequence and chaotic codes respectively. *M*_1_-*M*_4_ and *Ch*_1_-*Ch*_4_ are the 4 shifted versions of m-sequences and chaotic codes respectively. Each box (shown with pink color) represents temporal shift of 0.088 second (8 bits) ahead with respect to previous one.

The m-sequence and chaotic codes were presented at the rate of 90 Hz (each bit presented at 1/90 second). This is relatively higher presentation rate among the c-VEP studies as few studies have used presenting rates of between 80 Hz [18] and 120 Hz [9]. Four different versions of m-sequence and chaotic codes were generated by shifting the original code by eight bits that was temporally equal to almost 0.088 second (as shown in Fig 5). The circularly shifted versions of m-sequence and chaotic codes are shown by *M*_1_ − *M*_4_ and *Ch*_1_ − *Ch*_4_ respectively.

**Fig 6:**
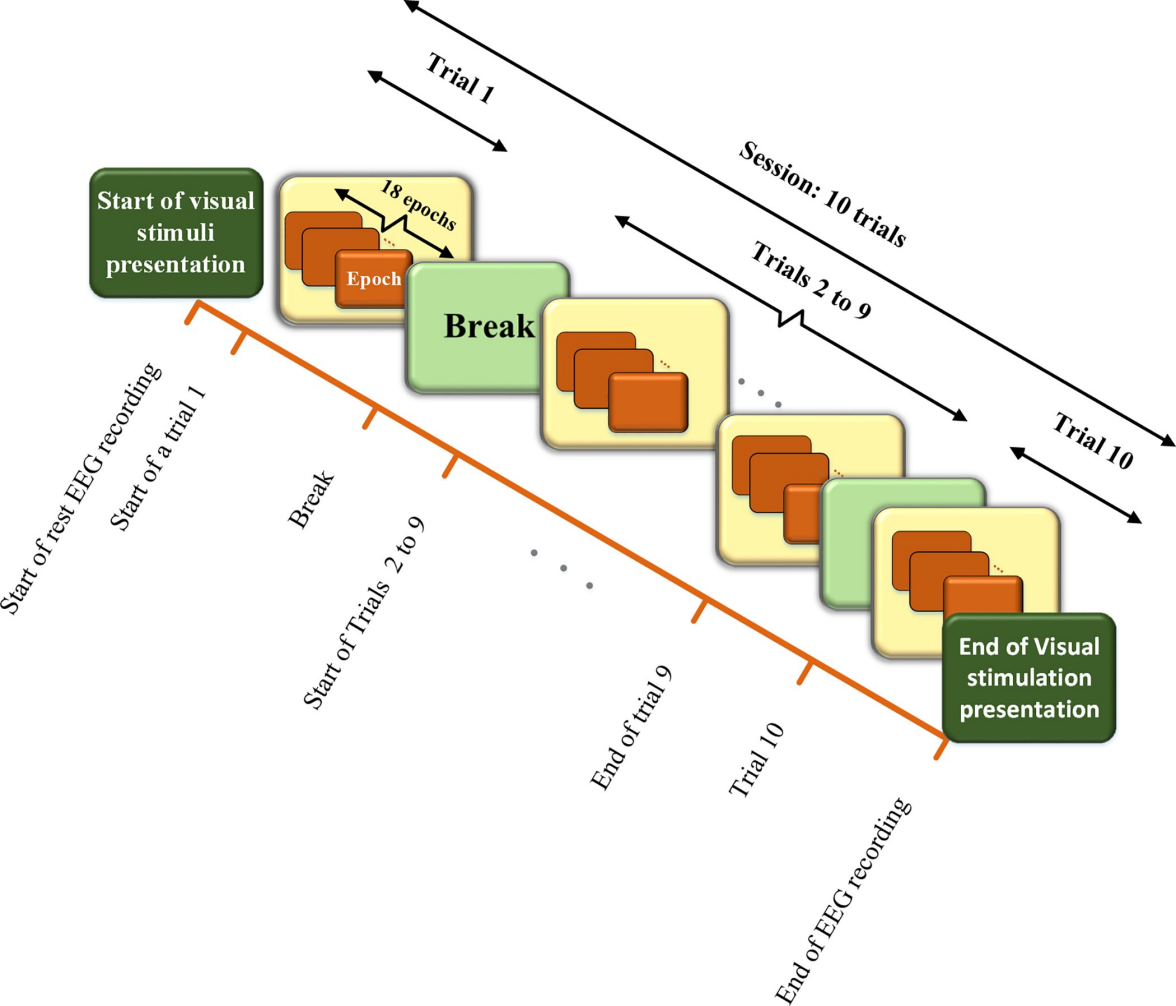
The stimuli presentation diagram for single session: A single session consisted of 10 trials presenting m-sequence or chaotic code visual stimuli. Each trial had 18 consecutively presented epochs. Each epoch presented a single visual stimulus code. Each trial was followed by 2 second break time. As there were 4 m-sequence (*M*_1_ − *M*_4_) and 4 chaotic codes (*Ch*_1_ − *Ch*_4_), each subject had total 8 sessions of stimulus presentation (see Fig 7 and text for details).

The stimuli specifications are presented in Table 1. Each code presentation duration time was 0.344 seconds (single epoch). It was presented 18 times in each trial (6.2 second). One session (90 second) of stimuli presentation consisted of 10 trials in which 2 second break was considered in between the trials. Supporting data files S1 Video and S2 Video recorded by Canon 750D DSLR Camera show playback videos of m-sequence and chaotic code respectively. Each video is approximately 17 seconds of total duration and has two trials with 2 seconds break in between. Stimulus presentation started 10 seconds after the start of EEG recording. Fig 6 shows the stimuli presenting diagram for single session.

**Table 1:** The stimuli specifications for both m-sequence and chaotic codes.

Stimuli presentation rate	Epoch duration (Code)	Epoch repetition per trial	Trial repetition per session	Rest duration between trials	Total duration of single session
90 Hz	0.344 sec	18 times	10 times	2 sec	90 sec

#### Subjective fatigue evaluation

All the participants were asked to answer the self-reported questions that measured the amount of fatigue and un-comfortability of the presented stimuli after each session. For evaluation of fatigue we used VAS score [89]. Before the start of session, the subjects were guided to report their fatigue rate by considering their tiredness of gazing the stimuli and how much they felt uncomfortable with the stimuli. At the end of each session, the subjects were asked to give the score (VAS score) between 0 for no fatigue at all and 10 if they were extremely fatigued. For avoiding the effect of cumulative fatigue by the previously presented stimuli, we let the subjects to have rest time of 2 minutes duration in between the sessions and then if the subject answered ‘No’ to the question “Do you need more time for rest?” we continued to record another session.

**Fig 7:**
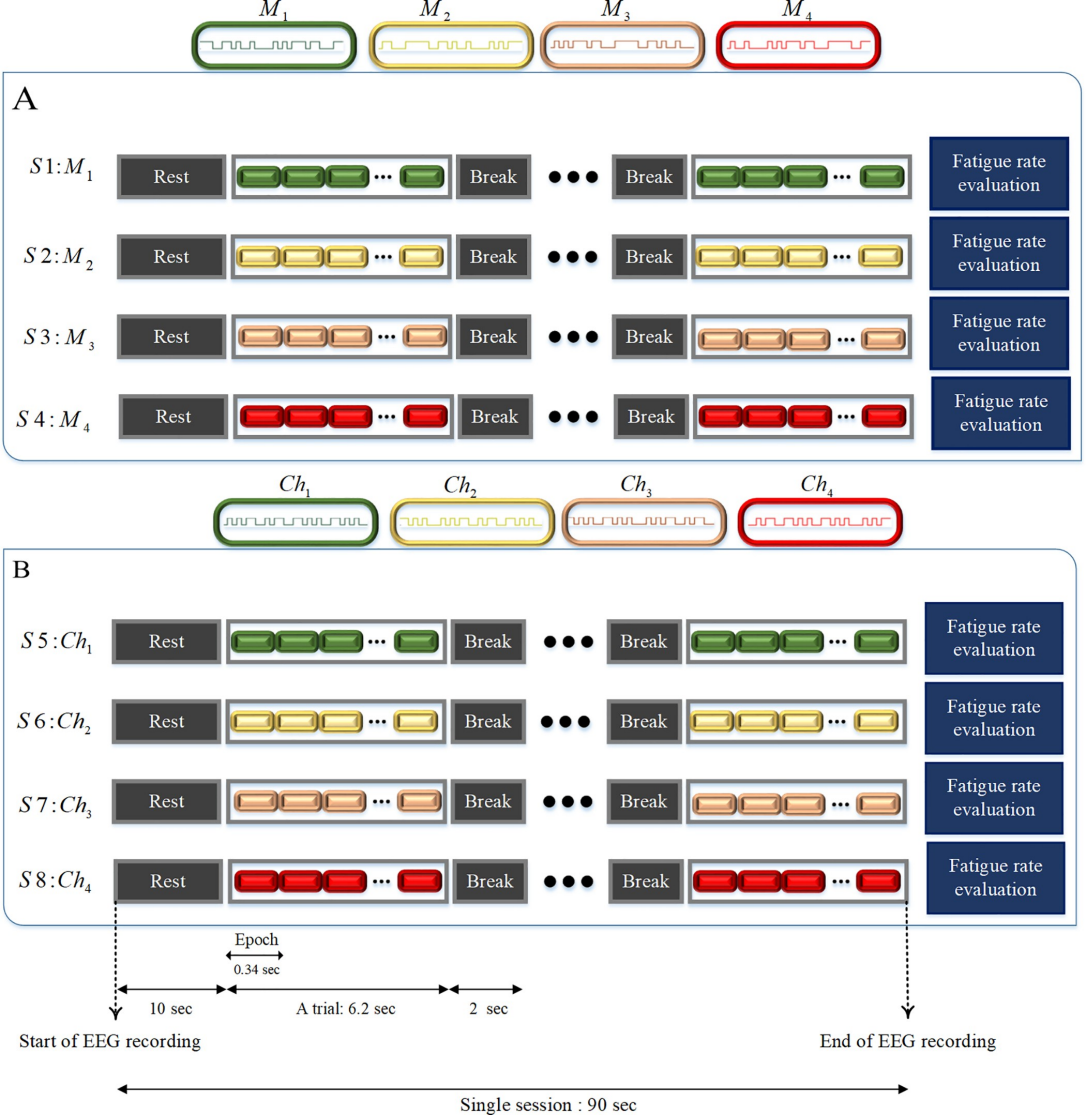
**Time sequences of activities of m-sequence (A) and chaotic code (B) presentation sessions. Each subject was presented with eight sessions.** Each session started with 10 second rest and EEG recording and consisted of 10 trials. Each trial consisted of 18 epoch of consecutive m-sequence or chaotic codes presented with 2 seconds break after each trial. At the end of each session subjective fatigue rate was evaluated. The order of presentation of eight sessions for each subject was random.

The order of presentation of the four shifted versions of m-sequence (*M*_1_-*M*_4_) and chaotic codes (*Ch*_1_-*Ch*_4_), comprising of total 8 stimuli codes, was random for all subjects. The random distribution of presentation sequence of each stimulus code in all 8 sessions helped to avoid the influence of bias caused by possible cumulative fatigue in our analysis. The time sequence of eight sessions of stimuli presentation with EEG recording and subjective fatigue evaluation is shown in Fig 7.

#### Signal recording setup

**Fig 8:**
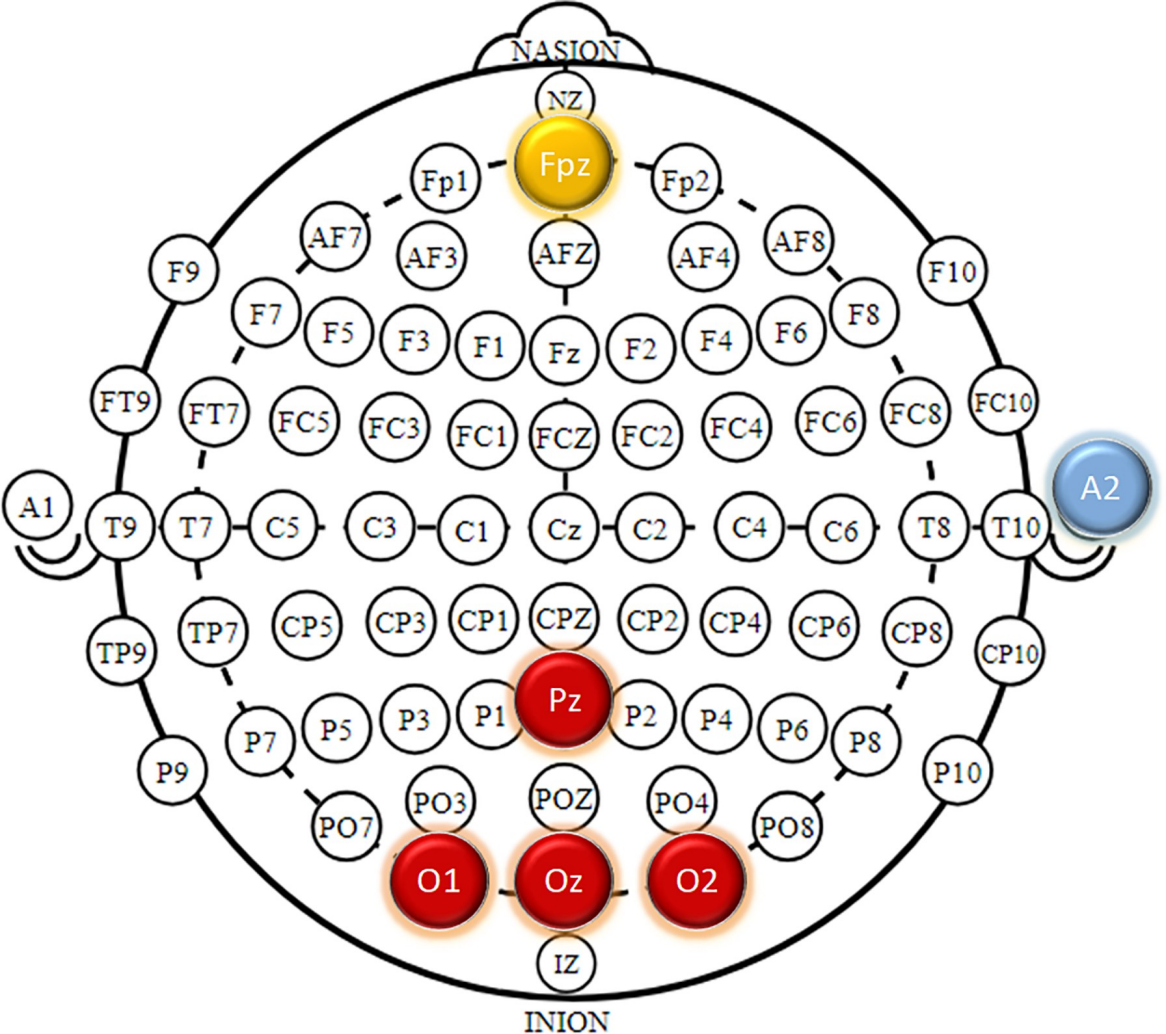
EEG recording electrodes placement according to 10–20 system. Four active g.laddy bird electrodes were placed in Oz, O1, O2 and Pz. A2 and Fpz were selected as the reference and ground electrodes respectively.

EEG signal was recorded using g.USBAmp with sampling rate of 4800 Hz. Four active g.lady bird electrodes were placed at Oz, O1, O2, and Pz positions on scalp where the visual evoked potentials such as c-VEP have maximum amplitude [11, 18]. Fpz and right earlobe were used as the ground and reference electrodes respectively as shown in Fig 8. An online band pass filter with cutoff frequencies of 0.05Hz and 120 Hz was applied.

**Fig 9:**
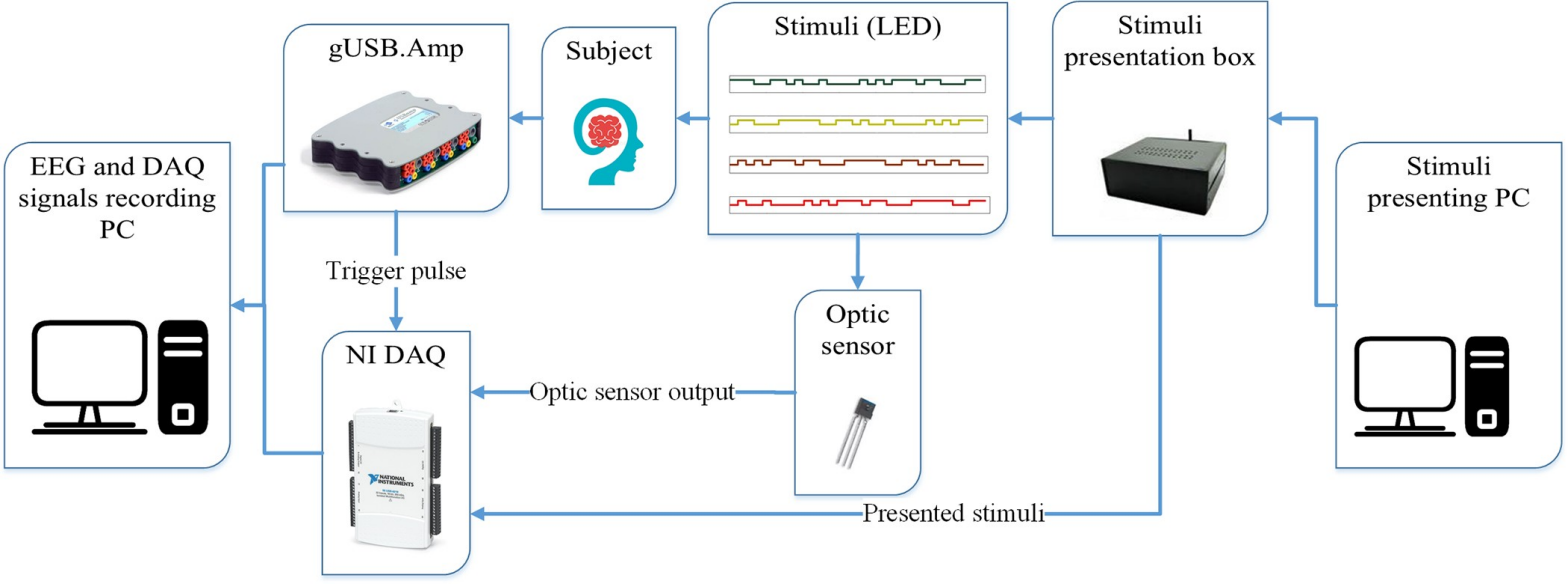
Signal recording set up: Stimuli were selected from stimuli presenting PC and sent to stimulator for presentation to subject via LED screen. NI DAQ was used to record the trigger pulse coming from g.USBAmp and optic sensor output and also the stimulator box. The data was sent to PC for further analysis.

All the stimuli were generated using MATLAB software (Release 2016b, The MathWorks, 193 Inc, Massachusetts, United States) and presented to a custom-made DAC board and LED driver (shown with stimulator in Fig 9). The LED used in this study was square shaped and red colored with size of 4 × 4 cm^2^ and was placed almost 70 cm from subject.

An optical sensor (Texas Instruments) was used to record the light of stimuli presented via LED and the National Instruments (NI) DAQ was used for recording the trigger pulse coming from digital Input-Output port of g.USBAmp signal recording amplifier that indicated the beginning of EEG recording. Additionally, Optic sensor output and analog output from LED driver were recorded in NI DAQ for synchronizing the stimuli presentation and EEG recording simultaneously. Finally the recorded EEG signal from g.USBAmp and NI DAQ were sent to a personal computer for further analysis. The onset of EEG recording and stimuli presentation were detected from recorded data in NI DAQ and the lag time between the two actions of start of EEG recording and start of stimuli presentation was identified. The EEG signal recorded after lag time was used for analysis. The beginning time of a trial was detected by the triggering pulse that came from LED driver at the beginning of trial.

The EEG recording and stimuli presentation set-up is shown in Fig 9. Details of the signal recording setup is reported in our previous study [90].

#### Preprocessing

The trigger pulse from g.tec and optic sensor output that was recorded with NI DAQ was extracted and used for detecting and extracting synchronized trials from EEG signals. The extracted EEG signal of individual trials was filtered by zero phase shift Butterworth band pass filter with cutoff frequencies of 2 and 40 Hz with the order of 8 and detrended for baseline correction. For each trial, the epochs that corresponded to each code were extracted and finally for each stimulus, 10 trials were extracted such that each trial contained responses to 18 consecutive epochs.

#### Feature extraction and target identification

Canonical correlation analysis (CCA) and spatiotemporal beamforming (STB) methods were used for feature extraction and target identification. For evaluation of feature performance, 10-fold cross-validation was used for the verification of above mentioned methods. This meant that all trials of a subject were divided into 10 folds; 9 folds were used as training data set and the remaining one fold was used as testing data. There were 10 trials for each of the four shifted codes every time when a single trial was tested during 10-fold cross-validation. Finally, the mean of 10 accuracies of target identification were reported as the final value of accuracy for each subject. All procedures were carried out for the responses to m-sequence and chaotic codes separately.

#### Canonical Correlation Analysis (CCA)

The CCA is a multivariable data processing that reveals the underlying correlation existing between the two multidimensional variables by maximizing the correlation of linear combination of two variables [91]. This method has been successfully used for the analysis of visual evoked potentials such as SSVEP [2, 11, 91]. CCA attempts to find the two vectors of *W*_*x*_ and *W*_*y*_ called as the canonical correlation vectors for the two multidimensional variables *X* and *Y* that maximize their canonical variant *x* and *y* which is defined respectively by *x* = *X*^*T*^*W*_*x*_ and *y* = *Y*^*T*^*W*_*y*_.

*W*_*x*_ and *W*_*y*_ derived by maximizing the correlation coefficient ρ:

$$\max_{W_{x},W_{y}}p(x,y)=\frac{E(x^{T}y)}{\sqrt{E(x^{T}x)E(y^{T}y)}}=\frac{W_{x}^{T}XY^{T}W_{y}}{\sqrt{W_{x}^{T}XX^{T}W_{x}*W_{y}^{T}YY^{T}W_{y})}}$$ (2)

In this study, the *X*^*m*×*n*^ is template and *Y* is the responses. In this study the matrices of *X* and *Y* are defined as matrices *s*^*m*×*n*^ and *T* respectively where *m* denote the number of channels and *n* is the number of samples in each epoch.

**Fig 10:**
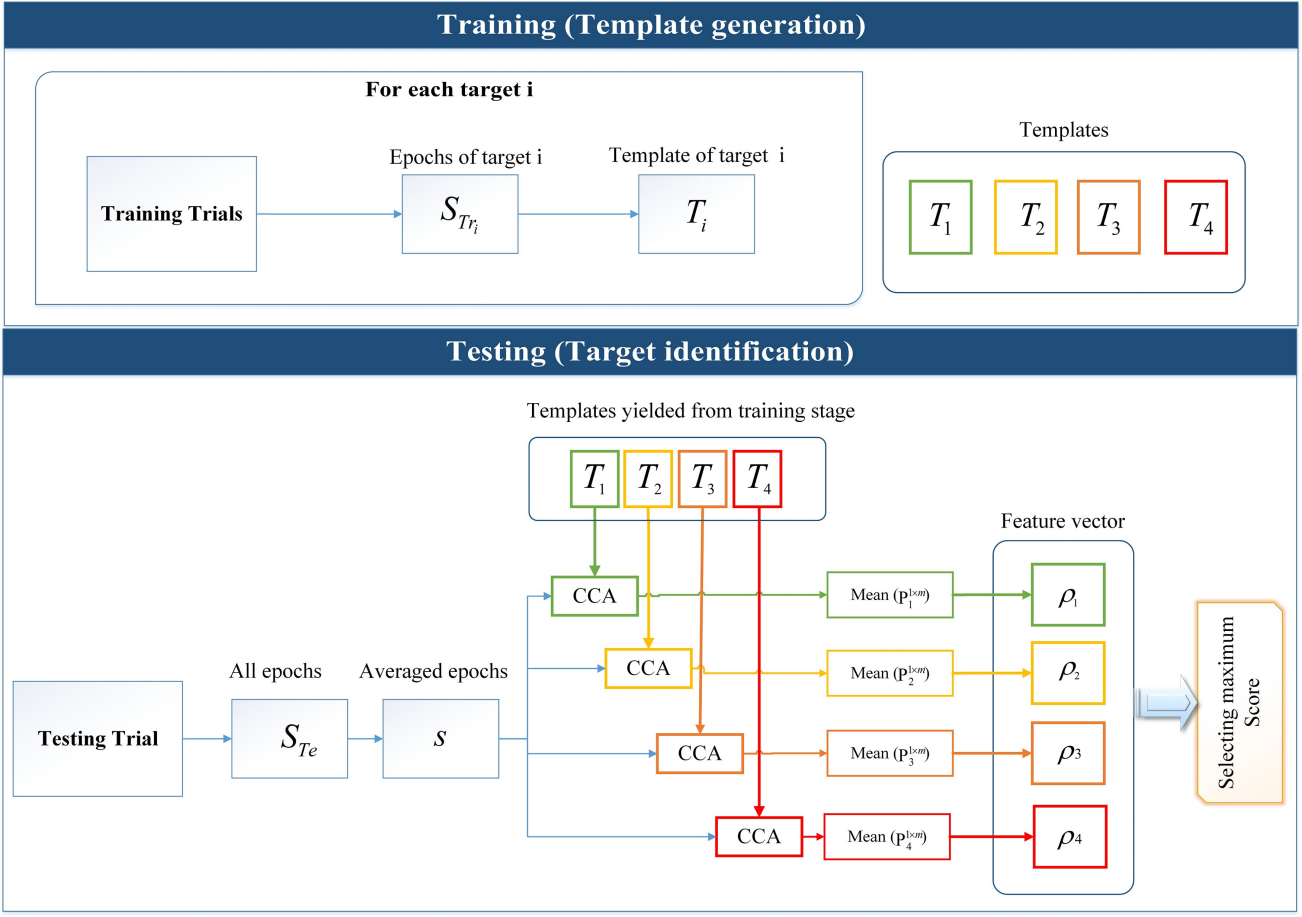
Schematic representation of using CCA for template generation and target identification. Template generation included extraction of epochs for each target and averaging them to generate templates. Target identification included extraction of epochs for testing trial and calculation of the canonical correlation of generated templates from training stage and averaged epochs for generating the feature vector and finally the maximum value of feature vector were selected.

The steps of using CCA for feature extraction and target identification are as follows and also shown in Fig 10.

#### Template generation

1. Extraction of the epochs in training data set $S_{Tr_{i}}^{k\times m\times n}$ where *i* = [1 2 …4] (the indices of *i* represent the *i* th target in m-sequence and chaotic codes separately) and *k* is the total number of epochs in training dataset.
2. Averaging $S_{Tr_{i}}^{k\times m\times n}$ over *k* epochs to generate the $T_{i}^{m\times n}$ which is then used as a template.

In online applications of code modulated BCIs, the templates are generally obtained for a single delay of code as a calibration target and the templates for other targets are obtained by shifting the original template [10]. In this study which was an offline study, due to the accessibility to training data set, we preferred to obtain the templates for each targets separately and therefore discontinuity introduced by circular shifting was prevented and even miniature differences between the templates were taken into consideration.

#### Target identification

1. Extraction of each target epochs of the test trial $S_{Te}^{r\times m\times n}$ where *r* is the number of epochs in the single trial.
2. Averaging $S_{Te_{i}}^{r\times m\times n}$ over *r* epochs to yield the matrix $s_{i}^{m\times n}$
3. Calculation of the canonical correlation of templates $T_{i}^{m\times n}$ and $s_{i}^{m\times n}$ to achieve the correlation vector *P*_*i*_^1×*m*^.
4. Calculation of the mean value of correlation vector *P*_*i*_^1×*m*^ to create the feature vector.
5. Selection of the maximum value of feature vector *ρ*_*i*_.

#### Spatiotemporal beamforming

STB was initially used as the spatial filter for analyzing the radar and sonar data [92]. STB has also been used in EEG signal processing for source localization [93] and optimal estimation of ERP sources [94]. The extended form of beamforming was introduced as a STB for single trial detection of evoked potentials from meaningful stimuli (N400) [95]. Recently the researchers in VEP based BCI have used this approach for decoding the message of each stimulus from synchronized EEG with it, such as P300 based BCI [96], SSVEP based BCI [97, 98] and c-VEP based BCI [9, 99].

**Fig 11:**
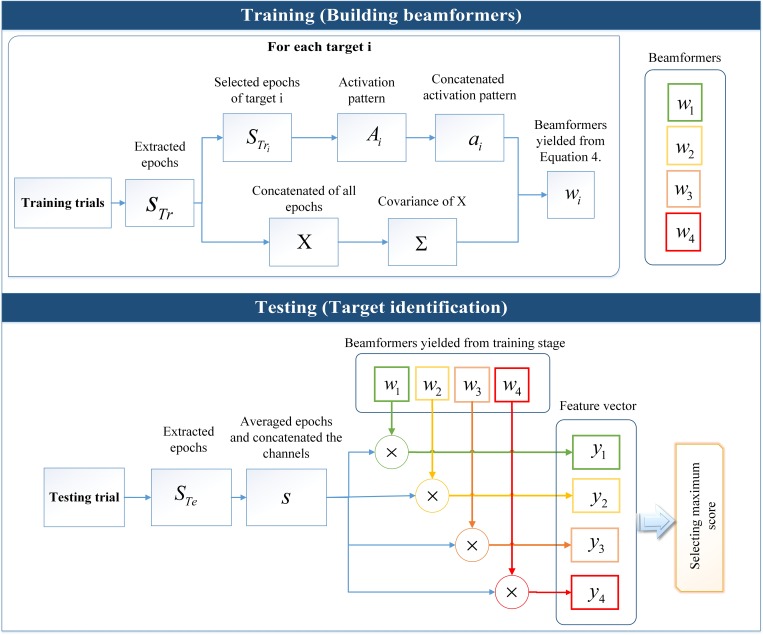
Schematic of representation of using STB for building beamformers and target identification. Building beamformers included extraction of epochs for all the targets and generation of the activation patterns for each target and in parallel calculation of covariance matrix of concatenated epochs. The beamformers were calculated from Eq 4. The target identification included multiplying the beamformers with concatenated channels of averaged epochs in testing trials for the generation of feature vector and selecting the maximum score.

The procedure for using STB is described in following steps and all procedures are shown in Fig 11 [99].

#### Building beamformers

1. Extraction of all the epochs of all the targets in training trials to create the matrix, *S*_*i*_^*h*×*m*×*n*^, where *h* is the total number of epochs of all the targets acquired from training trials data.
2. Extraction of the epochs corresponding to each target in training data ${S_{Tr_{i}}}^{k\times m\times n}$ where *k* is the epoch’s number in training trial data for each target.
3. Generation of the spatiotemporal activation patterns for each target *A*_*i*_^*m*×*n*^ by averaging ${S_{Tr_{i}}}^{k\times m\times n}$ over *k* epochs.
4. Concatenation of the rows of *A*_*i*_^*m*×*n*^ and generating the vectors of *a*_*i*_^1×*mn*^.
5. Generation of the *X*^*h*×*mn*^ by concatenating the channels in *S*_*Tr*_^*h*×*m*×*n*^.
6. Calculation of the covariance matrix of *X* for generating Σ^*mn*×*mn*^.
7. Generation of the beamformers *w*_*i*_^1×*mn*^ from:

$$w_{i}=\frac{\sum^{-1}a_{i}}{a_{i}^{T}\sum^{-1}a_{i}}$$ (3)

The linearly-constrained minimum-variance (LCMV) beamformers were calculated by using the Lagrange multipliers method under constraint $a_{i}^{T}w_{i}=1$ (3).

Note that due to the accessibility to training data, the activation patterns for each target were obtained separately for each target such as generating templates for each target in CCA method.

#### Target identification

1. Extraction of all epochs of testing trial $S_{Te}^{r\times m\times n}$ where *r* is the number of epochs in the testing trial.
2. Averaging *r* epochs and concatenating the channels of the averaged signal to generate *s*^1×*mn*^.
3. Calculation of *y*_*i*_ = *sw*_*i*_ where *i* = [1 2…4].
4. Selection of maximum score of *y* in feature vector.

#### Statistical analysis

The averaged VAS scores of each stimulation (m-sequence or chaotic codes) were calculated by averaging the scores across 4 sessions (shifted versions of codes) for each subject.

For the evaluation of subjective fatigue between the m-sequence code and the chaotic code groups, the averaged VAS scores were used and for comparison, the results were expressed as mean ± SE. For analysis of within group changes, repeated measures ANOVA for m-sequence and chaotic code was carried out separately on the individual VAS scores. A Greenhouse-Geisser correction with a significance level *α* = 0.05 was employed for analysis of within group changes in VAS scores for m-sequence code and chaotic group VAS scores. Then the post hoc analysis with Bonferroni correction was used for each pair comparison within the m-sequence and chaotic code groups while *α* set as 0.008.

Wilcoxon signed ranks test was employed for the analysis of the accuracy results yielded from 10-fold cross-validation and also comparing the accuracy changes between CCA and STB results over the stimulation time of 0.344 seconds (single epoch) to 6.2 seconds (18 epochs, single trial), the threshold was set at *α* = 0.05 for these analyses.

## Results

**Fig 12:**
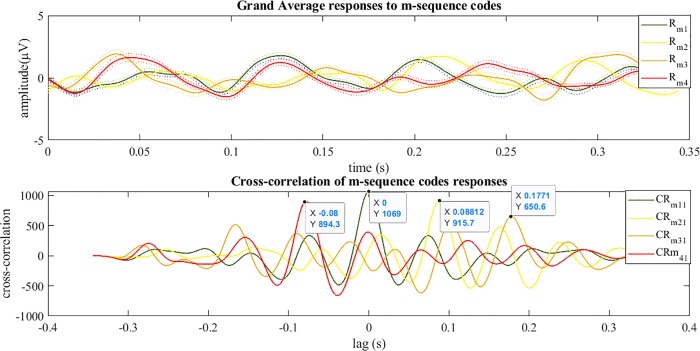
Grand average and cross-correlations of evoked responses to m-sequences. The grand average responses to codes *M*_*i*_ (*i* = 1:4) is shown with waveforms of $R_{m_{i}}$ their corresponding standard errors are shown with dotted plots (top) and the auto-correlation of response $R_{m_{1}}$ and its cross-correlation with the responses $R_{m_{i}}(i=2:4)$ is shown with the waveforms of $CR_{m_{i}}$ (bottom). The delay between responses could be decoded from cross-correlation waveforms where they are maximum.

**Fig 13:**
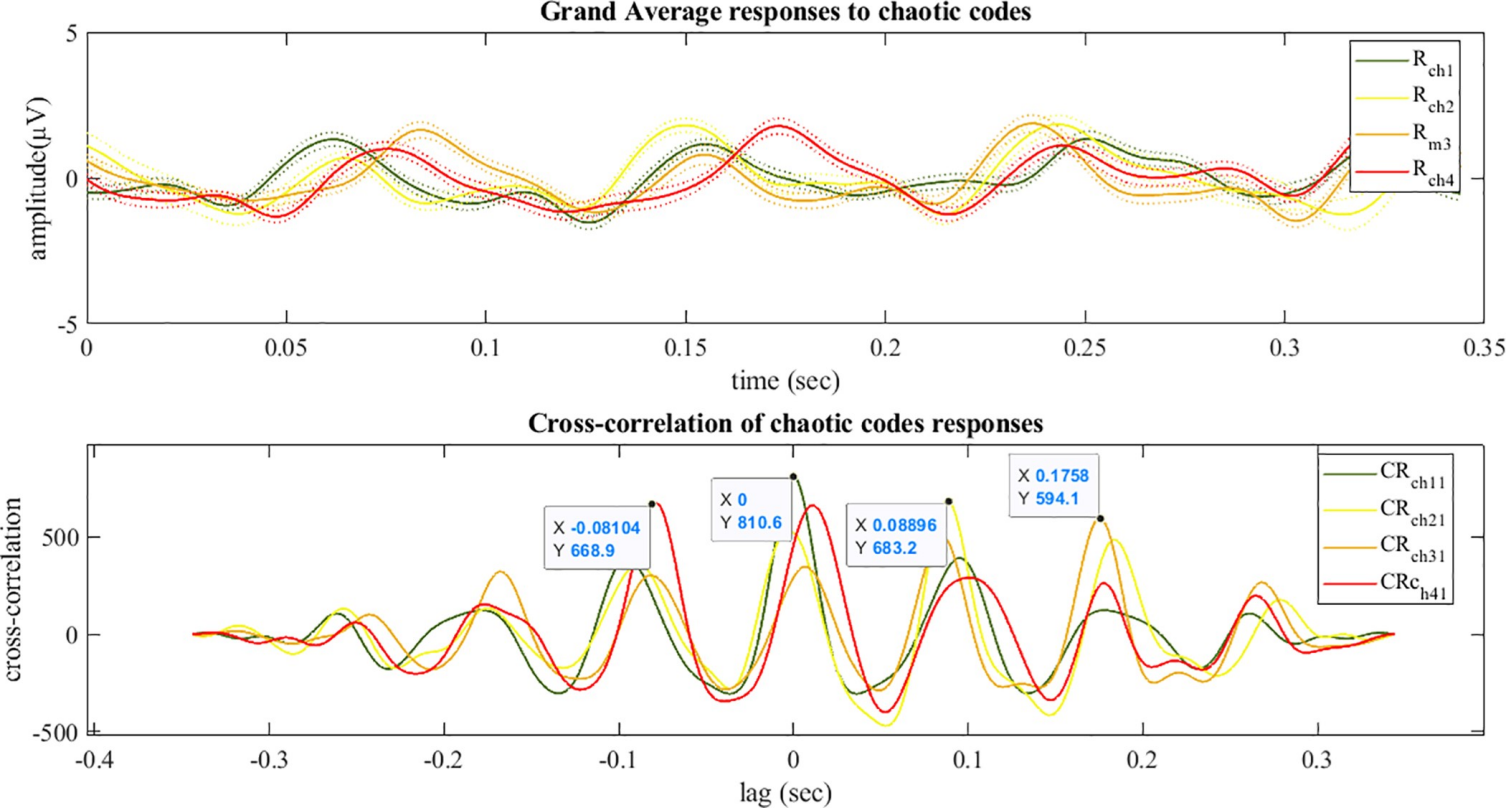
Grand average and cross-correlations of evoked responses to chaotic codes. The grand average of responses to codes *Ch*_*i*_ (*i* = 1:4) is shown with waveforms of $R_{{ch}_{i}}$ their corresponding standard errors are shown with dotted plots (top) and the auto-correlation of response $R_{{ch}_{1}}$ and its cross-correlation with the responses $R_{ch_{i}}(i=2:4)$ is shown with the waveforms of $CR_{ch_{i}}$ (bottom). The delay between responses could be decoded from cross-correlation waveforms values (as shown) where they were maximum.

Figs 12 and 13 show the grand average of evoked responses to m-sequences and chaotic codes. The grand averages of response for each stimuli was calculated by averaging all epochs in 10 trails and then across all channels and finally averaged for all subjects. For illustrating the existing delay between the m-sequence responses, the auto-correlation of $R_{m_{1}}$ (response to *M*_1_) and its cross-correlation with other responses $R_{m_{i}}(i=2:4)$ are shown in Fig 12. The similar results for the chaotic codes responses are presented in Fig 13.

**Fig 14:**
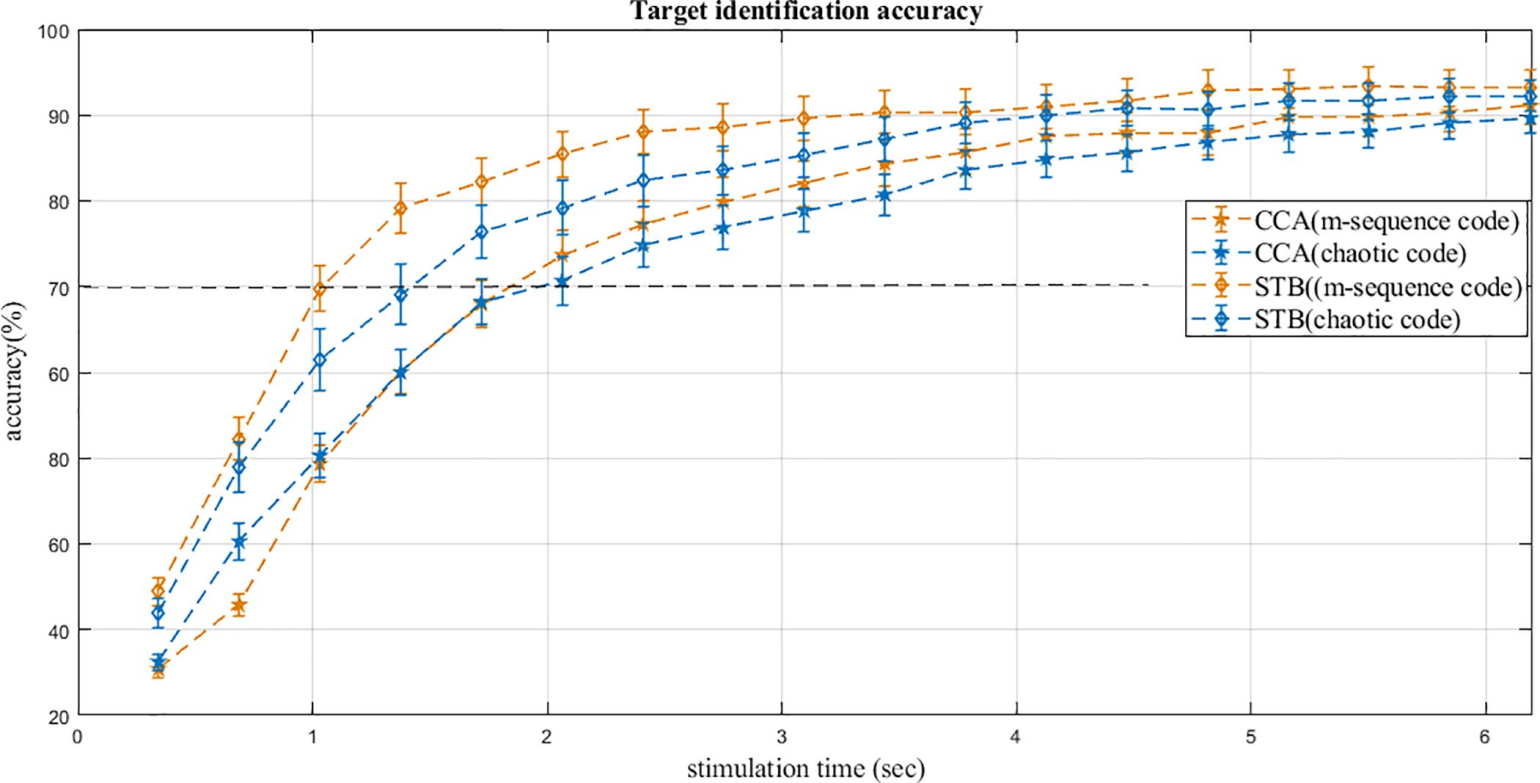
Accuracies of target identification for the m-sequence and chaotic codes obtained from 10-fold cross-validation with CCA and STB methods over stimulation time. Time duration for each epoch was 0.344 seconds and the total stimulation time for all 18 epochs was 6.2 seconds. The accuracy increased over stimulation time in both the methods. The dashed line shows that the STB is faster than CCA in reaching 70% accuracy.

Fig 14 shows the results of 10-fold validation over the stimulation time for m-sequence and chaotic codes. Increase in the stimulation times means the increase in the numbers of averaged epochs (code repetition) in test trials (from 1 to 18 epochs, from 0.344 to 6.2 seconds) for cross-validation.

The accuracies of target identification for 10-fold cross-validation for full stimulation time of a trial (6.2 seconds) are reported in Table 2. The maximum mean accuracy values of 93.6 ± 11.9% and 94 ± 14.4% were achieved with STB method for chaotic and m-sequence codes for all subjects respectively.

**Table 2:** Accuracies of target identification results of the 10-fold cross-validation for a trial for m-sequence and chaotic code for all subjects.

	m-sequence codes				chaotic codes			
	CCA		STB		CCA		STB	
**Subjects**	Mean (%)	SD (%)	Mean (%)	SD (%)	Mean (%)	SD (%)	Mean (%)	SD (%)
**S1**	92.5	2.8	100	0	97.5	0.6	100	0
**S2**	95	1.1	80	1.1	80	3.8	70	3.8
**S3**	42.5	4.2	20	9.4	60	3.0	55	1.1
**S4**	100	0	100	0	100	0	100	0
**S5**	67.5	4.2	95	1.1	90	3.0	100	0
**S6**	90	1.6	100	0	82.5	4.2	100	0
**S7**	95	1.1	100	0	82.5	1.4	95	1.1
**S8**	100	0	65	7.2	100	0	55	9.4
**S9**	100	0	100	0	100	0	100	0
**S10**	100	0	70	6.6	100	0	90	1.6
**S11**	97.5	0.6	100	0	87.5	1.7	100	0
**S12**	87.5	1.7	100	0	52.5	6.1	95	1.1
**S13**	100	0	100	0	100	0	95	1.1
**S14**	100	0	100	0	100	0	100	0
**S15**	85	3.0	95	1.1	82.5	4.2	100	0
**S16**	100	0	100	0	92.5	1.4	100	0
**S17**	70	2.5	85	1.6	67.5	5.6	60	4.4
**S18**	97.5	0.6	100	0	87.5	4.5	100	0
**S19**	90	1.6	100	0	87.5	1.7	100	0
**S20**	100	0	100	0	100	0	90	1.6
**S21**	87.5	3.1	100	0	75	6.9	90	1.6
**S22**	97.5	0.6	90	1.6	92.5	1.4	95	1.1
**S23**	100	0	100	0	100	0	95	1.1
**S24**	100	0	100	0	97.5	0.6	95	1.1
**S25**	100	0	100	0	97.5	0.6	100	0
**S26**	100	0	100	0	100	0	100	0
**S27**	100	0	100	0	100	0	100	0
**S28**	100	0	90	1.6	87.5	1.7	95	1.1
**S29**	72.5	6.1	95	1.1	87.5	1.7	100	0
**S30**	97.5	0.6	100	0	92.5	2.8	100	0
**S31**	97.5	0.6	100	0	97.5	0.6	100	0
**S32**	92.5	2.8	100	0	92.5	2.8	90	1.6
**S33**	100	0	100	0	97.5	0.6	100	0
**S34**	100	0	100	0	100	0	100	0
**S35**	100	0	100	0	100	0	100	0
**S36**	62.5	7.2	80	3.8	70	9.4	75	2.7
**S37**	97.5	0.6	100	0	87.5	3.1	95	1.1
**S38**	80	5.2	100	0	90	1.6	100	0
**S39**	57.5	8.4	75	2.7	72.5	4.7	90	1.6
**S40**	97.5	0.6	100	0	92.5	2.8	100	0
**S41**	92.5	1.4	100	0	87.5	3.1	95	1.1
**S42**	67.5	5.6	100	0	75	8.3	100	0
**S43**	100	0	100	0	100	0	100	0
**S44**	100	0	100	0	95	1.1	100	0
**Total accuracy**	Mean (%)	SD (%)	Mean (%)	SD (%)	Mean (%)	SD (%)	Mean (%)	SD (%)
	**91.13**	**13.8**	**94.0**	**14.4**	**89.5**	**11.7**	**93.6**	**11.9**

### Statistical analysis results

Significantly higher accuracy rates were obtained by Wilcoxon signed ranks test for STB method when we compared it with the CCA method accuracy rates at different stimulation times for both m-sequence and chaotic codes. Table 3 shows the comparison of accuracies results of CCA and STB methods for different stimulation times.

**Table 3:** Statistical results for accuracy values of paired t-test in the comparison between STB and CCA methods for m-sequence and chaotic codes.

		m-sequence code		Chaotic code	
Number of epochs	Stimulation time (sec)	Z	p value	Z	p value
**1**	0.344	-4.105	0.0005	-2.699	0.007
**2**	0.68	-5.324	0.0005	-2.974	0.003
**3**	1.03	-4.579	0.0005	-3.703	0.0005
**4**	1.37	-4.562	0.0005	-3.756	0.0005
**5**	1.72	-3.962	0.0005	-3.113	0.002
**6**	2.06	-3.752	0.0005	-3.228	0.001
**7**	2.40	-4.011	0.0005	-3.193	0.001
**8**	2.75	-3.469	0.001	-3.835	0.001
**9**	3.09	-3.211	0.001	-3.390	0.001
**10**	3.44	-2.984	0.003	-3.633	0.0005
**11**	3.78	-2.660	0.008	-3.384	0.001
**12**	4.12	-2.449	0.014	-3.363	0.001
**13**	4.47	-2.024	0.043	-3.368,	0.001
**14**	4.81	-2.314	0.021	-2.794	0.005
**15**	5.16	-2.312	0.021	-2.682	0.007
**16**	5.50	-2.350	0.019	-2.946	0.003
**17**	5.84	-2.321	0.020	-2.922	0.003
**18**	6.19	-1.916	0.05	-2.788	0.005

Wilcoxon signed ranks test showed no significant changes in the accuracy rates of STB method for the target identification of a single trial for the m-sequence and chaotic code groups (Z = -1.016, p = 0.31). Additionally, no significant results were observed when the accuracies of the m-sequence and chaotic codes groups were compared using CCA method for the single trial accuracies (Z = -1.204, p = 0.22).

### Between group fatigue analysis results

**Fig 15:**
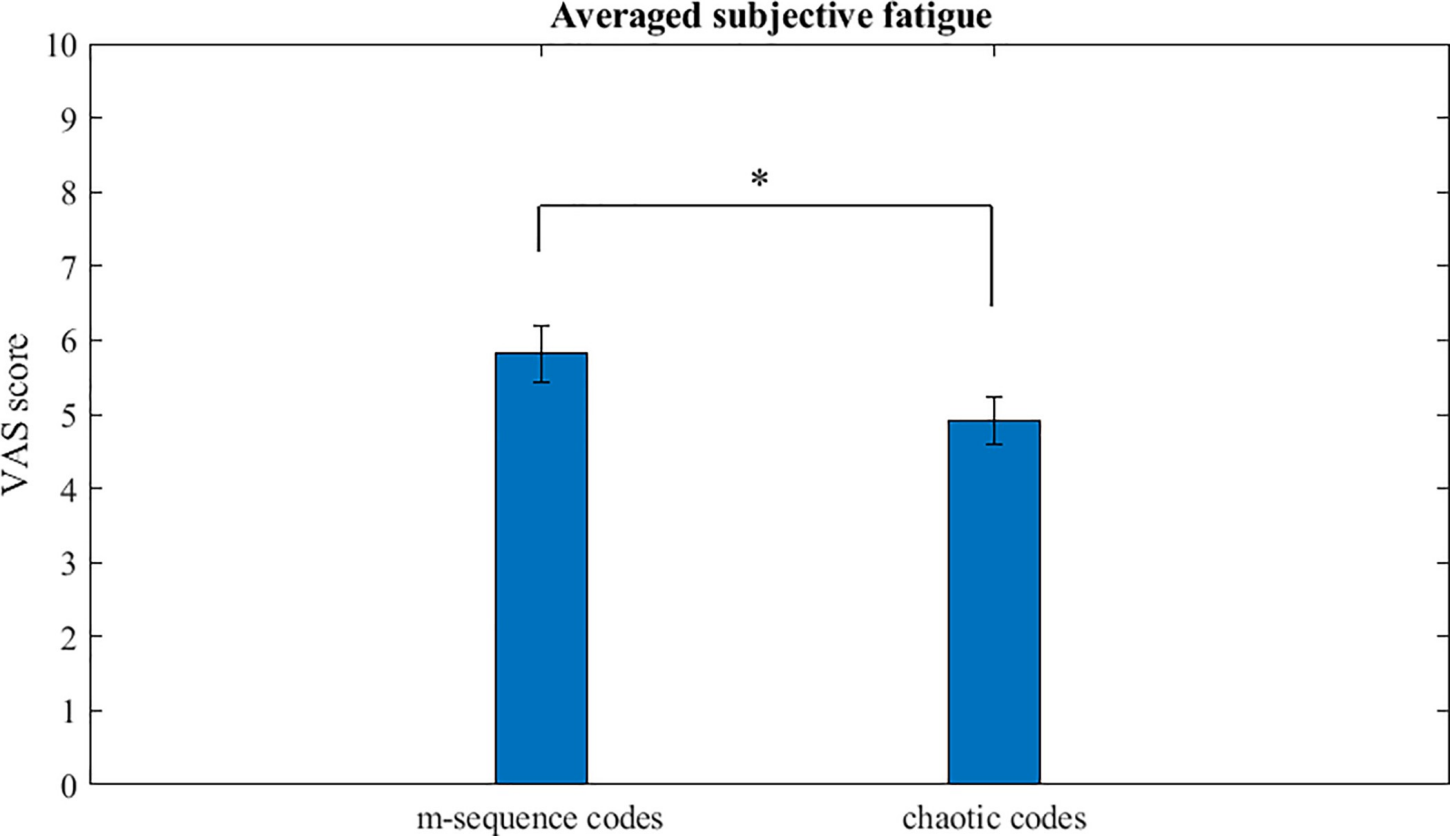
Averaged subjective fatigue scores of all m-sequence and chaotic codes of all the subjects. The chaotic codes VAS score was significantly lower than the m-sequence codes,*p = 0.0005, n = 44.

Chaotic codes resulted in significantly less VAS score (4.9076 ±2.1981) compared to the m-sequence codes (5.8152±2.6207) analyzed by paired t-test (t (43) = 4.054, p = 0.0005) as shown in Fig 15.

### Within group fatigue rate analysis results

No statistical changes were seen in the analysis of within group comparison of VAS scores with repeated measures ANOVA in m-sequence group (F (1.765, 79.434) = 0.754, p = 0.45).

Repeated measures ANOVA showed significant changes in the value of VAS scores in chaotic code (F (2.523, 113.521) = 5.345, p = 0.003). Post hoc analysis using Bonferroni correction with α = 0.008, showed significant differences between *Ch*_3_ and *Ch*_1_. Mean values of VAS scores of *Ch*_1_ and *Ch*_3_ were 4.58±2.32 and 5.19±2.34 respectively (p = 0.002). No significant results for other pairs of chaotic codes were seen.

**Fig 16:**
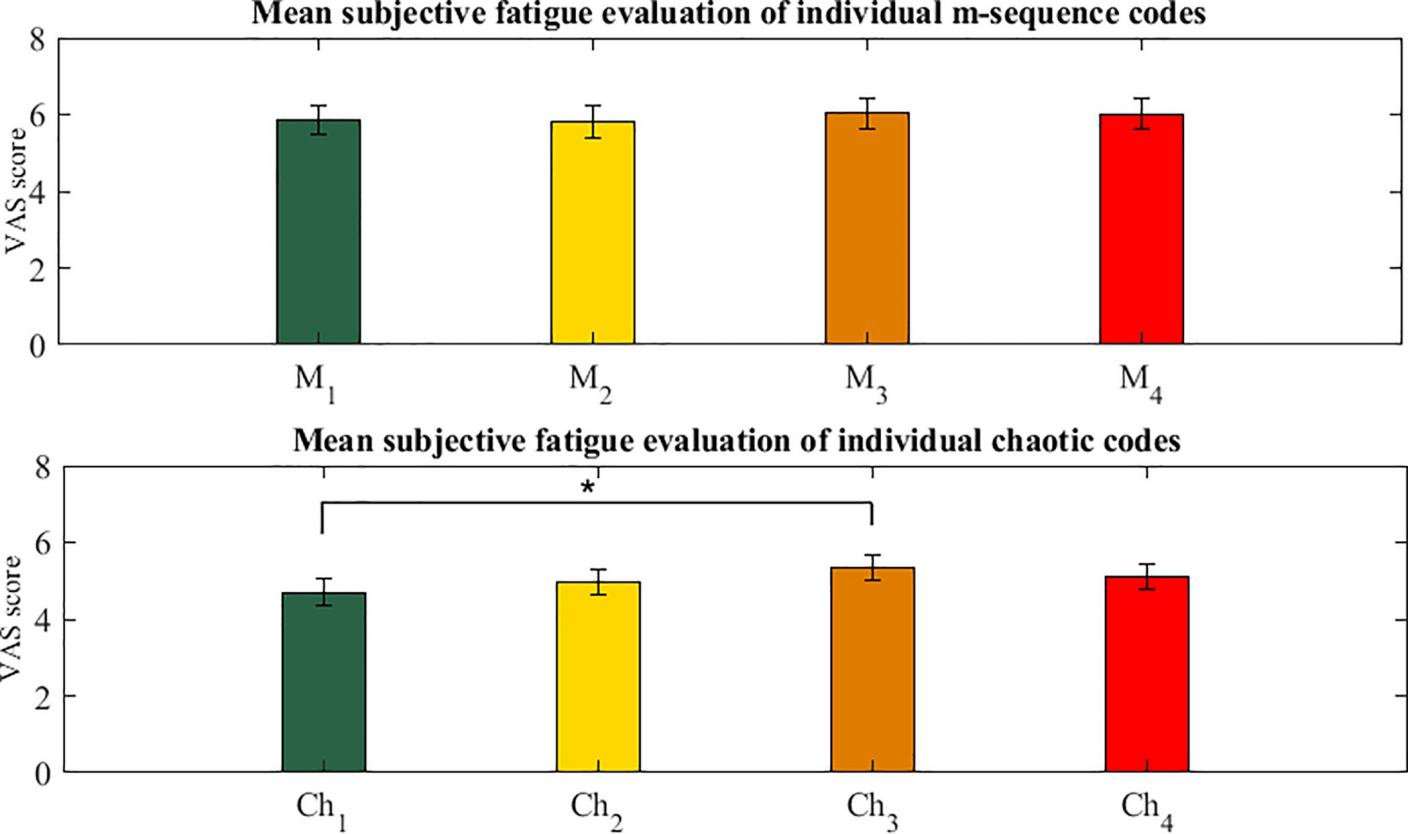
Subjective fatigue scores of individual m-sequence and chaotic codes. There was significant difference between VAS score of chaotic codes *Ch*_1_ and *Ch*_3_ (for each code n = 44 and *p = 0.002).

Fig 16 shows the subjective fatigue scores of individual m-sequence and chaotic codes.

## Discussion

In this study, we successfully used chaotic codes to evoke c-VEPs and found that the chaotic codes significantly reduced subjective fatigue compared to the conventional m-sequence code. We showed that the proposed code was able to evoke distinctive identifiable responses in EEG comparable with the m-sequence code that is currently employed in c-VEP response generation and code modulated based BCIs.

For the first time in code modulated based studies, chaotic codes presented as visual stimuli were identified successfully from their corresponding VEPs. The four shifted versions of m-sequence and chaotic codes used in this study had 8 bits circular delays ahead of pervious code. From Figs 12 and 13, it could be seen that the imposed delays of 0.088 seconds in between the presented chaotic code stimuli similar to the m-sequence code were preserved in their corresponding grand average responses. This delay time could be observed and detected in the peaks of the auto-correlation and cross-correlation of responses to each code (Figs 12 and 13 lower panels). The time when cross-correlation function was maximum determined the existing lag time between the intended stimuli and non-shifted version of codes. For example, the lag time between the response to *Ch*_1_ (zero bit shift) and *Ch*_2_ (8 bits shift) was 0.088 seconds which is represented as 8 bits between their corresponding stimuli (note that each bit shift is 1/90 seconds).

For the target identification of c-VEP to corresponding lag times in each group (m-sequence and chaotic code), we used CCA which is a common method for c-VEP analysis. We also used STB method recently introduced for the target identification in code modulated evoked potentials [9]. By increasing the stimulation time (increasing the numbers of epochs to be averaged), the accuracies of target identification increased; for m-sequence code, the total accuracies achieved were 91.13±13.8% and 94 ± 14.4% by CCA and STB methods respectively. For chaotic codes, the total accuracies of 89.5 ± 11.7% and 93.6 ± 11.9% were achieved by CCA and STB methods respectively (Table 2). The results of data analysis showed the m-sequence and chaotic codes in target identification results had no significant differences for both methods.

Our results for m-sequence and chaotic codes show that the total accuracy was over 70% in CCA method after approximately 2 seconds (6 epochs). Also, in STB method for m-sequence and chaotic codes after approximately 1 seconds (3 epochs) and 1.5 second (4 epochs) respectively, the total accuracy was 70% (Fig 14), which is acceptable in BCI applications [100].

Our results indicate that the STB method was significantly better than CCA method especially at the shorter stimulation time for m-sequence codes (Fig 14 and Table 3). However, for the longer stimulation time, STB method was comparatively more significant than CCA method for chaotic codes (Fig 14 and Table 3). In addition, the accuracy increased faster with STB compared to the CCA method (Fig 14). Therefore we can conclude that STB is faster than CCA as the 70% accuracy was achieved sooner with it. In a previous c-VEP based study, STB method has also been shown to be better than SVM method [9].

The most important result of our study is the significant reduction of subjective fatigue in the chaotic codes group compared to the m-sequence codes (Fig 15). Reason for higher subjective fatigue in the m-sequence group is the fact that while both the codes had broad band frequency spectrum, the spectral properties of m-sequence codes used in our study were more towards lower frequency spectrum (Fig 4) which causes more subjective fatigue and visual discomfort compared to the higher frequencies visual stimuli [69, 90] and have high risk of photosensitive epileptic seizures [101].

In addition, reduction of fatigue and visual discomfort as seen in the chaotic group was because of the fact that chaotic stimuli had higher frequency spectral distribution as shown in Fig 4. Frequency components higher than 30 Hz reduce the probability of occurrence of fatigue and visual discomfort because high frequency components are hardly visible and imperceptible to human eye [69].

Additionally, visual stimuli with excessive contrast energy at medium frequencies spectrum at the range of 10 to 30 Hz such as m-sequence codes used in our study can increase the eye discomfort level [75]. It is obvious from the comparison of spectral content of m-sequence code and chaotic code as shown in Fig 4 that the m-sequence code had more dominant peaks within the medium frequency range while the chaotic codes had more dominant peaks within the frequency component higher than 30 Hz. Considering the above reasons for the significant reduction of subjective fatigue by chaotic codes, we suggest their use for designing ergonomic c-VEP based BCI applications.

Another possible reason for the significant reduction of subjective fatigue with chaotic codes used in our study is the closeness of chaotic behavior to the 1/f spectral property [76, 77] which is observed in natural scenes and phenomena. It is widely reported that most of natural phenomena exhibit the 1/f type of spectral properties [75, 102–104]. Interestingly, visual system encoding is more efficient when encountering the stimuli with spatial and temporal patterns resembling 1/f amplitude spectral features [75, 105]. Visual stimuli with the above characteristics and patterns such as chaotic codes, generate sparse cortical responses in the receptive fields of neurons in the primary visual cortex [106]. As the hemodynamic responses mainly reflect the local field activity of neurons [107], the sparseness in the number of firing the neurons may lead to lesser demand for oxygenated blood and hence less fatigue.

fMRI and near infrared spectroscopy (NIRS) show that the oxygenation is more prominent when the visual stimuli are relatively uncomfortable [107, 108] as seen with the m-sequence codes that have pseudo-random behavior and flat wideband spectrum [78, 79] increasing the probability of discomfort level.

Our results of within group comparisons of individual VAS scores of m-sequences and chaotic codes show that the m-sequence (*M*_1_ − *M*_4_) did not cause significantly different fatigue level. However, in the chaotic code group, *Ch*_1_ code’s VAS score was significantly less than *Ch*_3_ (Fig 16). The significance value of this within group difference is very less compared to the overall difference in the fatigue level between the m-sequence and chaotic code group. We don’t have any explanation for this result and suggest further studies on chaotic codes in c-VEP based studies to find exact reason for it.

### Importance of chaotic visual stimuli and suggestions for future works

Researches during last few years have shown that in several areas of visual system, information processing involves dynamical and nonlinear processes as seen in retinal ganglion cells [109, 110], retina [111], lateral geniculate nucleus [104] and visual cortex [112]. In addition, spatial integration of information in retinal ganglion cells [109, 110] and colored visual stimuli processing of primary visual cortex [113] also involve nonlinear dynamics. Visual stimuli with chaotic dynamics involve not only primary visual cortex but also parietal-occipital and parietal areas of the brain [114]. We thus suggest use of chaotic visual stimuli for future c-VEP based studies as these conform to the biological reality of nervous system. Further research is also suggested for neural processes in visual cortex on mechanisms of lesser fatigue with chaotic dynamical stimuli.

The results of this study also suggest use of chaotic codes and nonlinear analysis as it may be the underlying nonlinear dynamics in chaotic stimuli that can be decoded better than conventional analysis method used for target identification.

As this study is the first of its kind in c-VEP based investigation, our limitation was that we didn’t study effect of change of logistic map parameter on target identification accuracy and subjective fatigue values. Therefore in the future studies, we suggest optimum parameters for generating chaotic code. We also suggest use of visual stimuli that are more close to the 1 /f spectral property.

Finally, as the results of our study show that chaotic visual stimuli are identifiable by CCA and STB methods and cause less fatigue compared to the conventional m-sequence codes, we suggest further c-VEP studies using these two new and other methods for designing better CDMA based BCI in future.

### Conclusion

This study for a first time examined chaotic code used for evoking c-VEP in CDMA based BCIs and compared the results with conventional m-sequence code widely used in code modulated BCIs. Our results show that the chaotic code was decoded successfully from recorded EEG responses and complied with the requirements needed for using it as a modulating code in the c-VEP generation. Better fatigue reduction was achieved by using chaotic code compared to the m-sequence code. We suggest use of chaotic code in c-VEP based studies for better application of BCI.

## Supporting information

S1 File m-sequence code presentation. Two consecutive trials of m-sequence code. Click here for additional data file. (MP4)

S2 File Chaotic code presentation. Two consecutive trials of chaotic code. Click here for additional data file. (MP4)
